# Placebo Effects in Modern Medicine: Mechanisms, Clinical Evidence, Limitations, and Future Directions

**DOI:** 10.7759/cureus.100612

**Published:** 2026-01-02

**Authors:** Mayar B Alnasralla, Basel H Nasralla

**Affiliations:** 1 Neuroscience, Royal Private English School, Fujairah, ARE; 2 General Surgery, AlSharq Hospital, Fujairah, ARE

**Keywords:** anxiety, classical conditioning, depression, ethical challenges, neurobiology, open-label placebo, pain management, patient expectations, placebo effect, psychology

## Abstract

This narrative review traces the historical background of placebo use in medicine, from its early role as a consolatory intervention prior to evidence-based practice to its scientific reframing with the emergence of randomized controlled trials and the pivotal contributions of mid-20th-century research. The article examines the mechanisms underlying placebo effects, focusing on neurobiological processes involving pain-modulating brain networks, endogenous opioid and dopaminergic pathways, and emerging evidence for hormonal and immune system involvement, alongside psychological mechanisms such as conditioning, patient expectations, and therapeutic engagement. Different types of placebo interventions, including placebo pills, sham procedures, and surgical placebos, are discussed to illustrate how treatment context and ritual contribute to symptom improvement. The methodological role of placebos in clinical trials is outlined, emphasizing randomization, blinding, allocation concealment, and outcome measurement as essential elements for distinguishing true treatment effects from non-specific influences. Clinical evidence of placebo effects is reviewed in pain management, including chronic and acute pain conditions, as well as in mental health disorders such as depression and anxiety. Case studies highlighting both successful applications and failures of placebo effects are presented, followed by a discussion of key limitations related to study design, response variability, and ethical concerns, including deception and withholding treatment. By integrating historical, mechanistic, methodological, clinical, and ethical perspectives that are often addressed separately in existing literature, this narrative review aims to fill an important gap by providing a cohesive and comprehensive synthesis of the placebo effect and its relevance to contemporary research and clinical practice, while clarifying its potential and limitations.

## Introduction and background

The placebo effect illustrates the powerful interplay between the mind and body in healing. Historically, placebos, derived from the Latin placēbō, “I shall please”, were used to comfort patients when no effective therapy existed [1]. By the 18th and 19th centuries, they were a common clinical tool, and with the advent of randomized controlled trials (RCTs), placebos became scientifically recognized as a standard for distinguishing true treatment effects from psychological and contextual influences [1-2]. Henry K. Beecher’s seminal work in the 1950s highlighted the role of patient belief in treatment outcomes, particularly for pain and post-surgical recovery, marking a turning point in medical appreciation of the phenomenon [3-4].

Neurobiologically, placebos engage brain networks involved in pain, emotion, and reward, including the anterior cingulate cortex, insula, thalamus, and periaqueductal gray, and activate opioid and dopaminergic pathways, with potential effects on immune and hormonal function [5-8]. Psychologically, conditioning, patient expectations, and the clinician-patient relationship shape symptom perception and therapeutic response [9-11,8]. Placebos take various forms, from pills to sham procedures and even surgeries, emphasizing the role of context and ritual in eliciting improvements [12-14].

Placebo effects are evaluated in placebo-controlled trials, where randomization, blinding, allocation concealment, predefined outcomes, and adequate sample sizes help isolate true treatment effects from non-specific influences such as natural recovery, patient expectations, and study interactions [15-17].

Clinically, placebo responses are evident in chronic and acute pain [18-20], depression and anxiety [21-24], and functional disorders like irritable bowel syndrome and osteoarthritis [25,26]. While methodological variability, individual differences, and ethical concerns pose challenges [27-37], open-label placebos offer an ethically acceptable approach to harness these effects [26,37]. Emerging research on genetic influences, immune modulation, and the microbiome-gut-brain axis further expands our understanding of placebo mechanisms and their potential clinical applications [31,38-39].

The aim of this narrative review is to provide a comprehensive overview of the placebo effect, integrating current knowledge and addressing gaps in the literature. Although many studies have explored specific aspects, there is a lack of synthesis connecting mechanisms, clinical relevance, and future research directions. This review fills that gap, offering insights to guide patient-centered care and inform further studies.

Historical background of placebo effects

The use of placebo treatment in medicine dates back further than most people think. Prior to the introduction of evidence-based medicine, doctors commonly (and, at one point, outright intentionally) prescribed medication they knew was therapeutically useless. The term placebo has the Latin origins "placēbō", which translates to "I shall please", which initially began as a form of comfort when nothing better could be offered to patients [1].

Placebos were almost standardized in the 18th-19th-century clinical practice. They would be used by doctors to soothe patients while the body healed itself. This reasoning was mostly intuitively derived; they had little understanding of how mind and body interacted and saw it as a practical choice: a safe pill was superior to no intervention when there was no treatment [1].

However, during the 20th century, with the rise of RCTs, placebos were reframed scientifically. By providing a standard for comparison, placebos helped distinguish between true pharmacological effects of a drug from psychological and placebo-related factors [2].

In the journey that placebo research takes, the pivotal turning point can be found in the 1950s, when Henry K. Beecher’s study “The Powerful Placebo” focused attention on the role of patient belief in the results of treatment of pain and post-surgical recovery by showing that as much as a third of the effects of treatment could be attributed to the placebo effect [3]. Even though his methodology and conclusions were later criticized by other researchers, Beecher's work was inevitably the central point in the shift of the medical community’s attitude toward the phenomenon of the placebo effect.

That has opened the subject since then. Techniques that have come along, like advanced scanning devices, neurobiology, and clinical psychology, have peered beneath the surface of what goes on in a placebo response. They have pushed aside imprecise psychological explanations and started to attribute the effect to more specific biological processes [4].

The topic that started as an attempt to offer consolation to patients has now turned into a well-established field of study and research. From considering the mind to be separate from the body to owning up to that it has notifiable effects on physical health, this advancement helped shape a deeper understanding of the connection between the mind and body in healing.

## Review

Mechanisms of the placebo effect

Neurobiological Mechanisms

From a biological standpoint, the placebo effect stimulates a complex sequence of reactions in the brain that influence not only pain perception but also emotions and bodily sensations [5]. Functional neuroimaging studies have consistently shown that placebo treatments alter activity within pain-related brain networks, particularly in regions such as the anterior cingulate cortex, insula, thalamus, and periaqueductal gray, all of which play central roles in the modulation and control of pain [6]. When activated, these areas facilitate the release of endogenous opioids, which are our body's natural analgesics and reduce the feeling of pain to produce analgesia [5].

While often attributed to the opioid system, recent literature suggests that placebo responses activate additional neurotransmitter pathways, including dopamine pathways in the brain, which are intrinsically linked to the brain's reward and motivational system. This activation supports the notion that when we expect to improve, we can regulate our mood using dopaminergic systems [7]. The reward and anticipation become part of the healing process in and of themselves and facilitate symptom relief and improvement. In addition to changes in neurotransmitter function, emerging evidence proposes that placebo effects may also have an effect on immune function and processes regulating hormones. This suggests that the body is affected holistically when treated under placebo conditions [8].

Psychological Mechanisms

Psychologically, the placebo effect depends on a variety of interrelated processes that together shape how a person experiences healing. The most essential elements include Pavlovian conditioning, patient expectations, and the quality of therapeutic encounters. Firstly, Pavlovian conditioning (sometimes called classical conditioning) explains how patients develop automatic, learned responses to specific treatment cues. For example, if a patient has symptom relief every time they take a pill, their brain begins to connect the taking of the pill, regardless of what is in it, to the treatment itself, with some healing. Over time, even a placebo can create responses in the body that trigger physiological changes [9]. The influences often operate outside of conscious awareness to shape how the brain interprets the treatment context.

Patient expectations play a central role in placebo responses. The anticipation of relief influences how individuals perceive pain, anxiety, and other symptoms, shaping the overall therapeutic outcome. When a patient truly believes the treatment is going to help them, the positive expectation reduces the perception of symptoms [10]. This comes from previous experiences and is influenced by information given by healthcare professionals generally [11].

Finally, therapeutic engagement itself is an important psychological factor. It involves the clinician-patient relationship, in which empathy, trust, and positive communication play a crucial role. A supportive and caring therapeutic environment can foster placebo results by providing safety and hope, which may further activate biological healing factors [8]. The clinician's wording, tone, and manner also help shape the patient's mindset and expectations, which could further strengthen the placebo effect as discussed earlier.

Together, these psychological processes reveal how the mind engages in the interpretation and response to treatment contexts and underscore a critical experiential triage that weaves together belief, learning, and social factors. This connection confirms that placebo effects reveal important psycho-emotional factors that influence (and often mediate) physical health.

Types of placebo treatments

Placebos are of many different types, and with each is its own interpretation as to how the placebo effect manifests itself. The most known is probably the placebo pill, a harmless substance that patients believe is an actual active medication. Although these pills appear to be rather simple, they have demonstrated surprisingly strong effects across various conditions, such as pain relief and mood improvement (discussed in more detail later) [12].

However, placebos do not only come in pill bottles. Sham procedures mimic real medical interventions without delivering any therapeutic element. These include placebo injections, devices, and sometimes even fake surgical procedures where no actual surgery occurs [13]. Placebo surgery, in particular, is a compelling example of the extent to which the ritual and context of treatment contribute to outcomes. Why? Because studies have found that patients subjected to sham surgeries obtain reported improvements that are comparable to those of patients who were provided the actual procedure in certain cases [14]. This phenomenon clearly clarifies that the healing process is highly integrated into the mind and context, as well as the actual intervention itself.

Each placebo type offers individual insight into the complex mechanisms of the placebo effect. Building on this understanding, important questions arise, such as how these effects can be incorporated into clinical care effectively.

Clinical trials and placebo controls

Placebo-controlled trials are widely recognized as the gold standard for evaluating novel medical therapies. By including a placebo group, researchers can isolate the specific effects of a drug or intervention from non-specific influences. These non-specific factors include natural recovery over time, psychological influences such as patient expectations and conditioning, and the additional attention and interactions participants receive within the study context. Properly designed placebo-controlled trials, therefore, allow clinicians and researchers to distinguish true therapeutic effects from these confounding influences, ensuring that observed outcomes reflect the efficacy of the intervention itself rather than extraneous factors [15].

To achieve valid results, careful trial design is essential. Central to this is randomization (to allocate individuals to either treatment or placebo groups without bias) and blinding (to limit knowledge of who is receiving which treatment for both the participant and the investigator). These components of the trial design reduce bias and increase the validity of generalizing from the findings. If blinding or allocation concealment is disrupted, it increases the likelihood of an inadvertent influence on the outcomes, often leading to overestimated treatment effects [16].

Other design features further enhance the rigor of randomized controlled trials. Proper allocation concealment protects against selection bias during participant enrollment, ensuring that group assignments cannot be anticipated or manipulated. Clearly predefined outcome measures limit the risk of selective reporting, allowing researchers to evaluate treatment effects as originally planned. Adequate sample sizes, combined with appropriate statistical analyses, increase the study’s power to detect true differences between groups. Collectively, these design strategies strengthen the internal validity of trials and support the generalizability of findings beyond the study population [17].

Placebo effect in pain management

Chronic Pain Studies

Placebo analgesia in chronic pain syndromes can persist for weeks or even months. This is especially relevant for conditions such as fibromyalgia and osteoarthritis, where conventional treatments often provide only limited relief and may cause side effects. Placebo responses in these patients are comparable in strength to those seen in healthy individuals, with both showing reductions in pain severity [18,19].

Despite these promising outcomes, integrating such strategies into standard care remains challenging. Not all patients experience lasting benefits, and most current evidence comes from small or short-term studies. Larger, long-term trials are needed to determine how these placebo approaches can be reliably incorporated into chronic pain management.

Acute Pain Studies

In acute pain, such as after dental surgery, placebo interventions can produce measurable pain relief even without active medication. This effect is evident in the early postoperative period, as patients report reduced pain a few hours after administration of a placebo. Such findings highlight that placebo responses can occur quickly in acute pain settings, although their precise duration remains unclear [20].

Effects of the placebo on mental health

Placebo effects are particularly evident in psychiatric conditions, where subjective experiences and patient expectations strongly influence outcomes. Among mental health disorders, depression and anxiety have been the most extensively studied, revealing that placebo interventions can produce measurable symptom relief. However, the strength and stability of these effects vary across individuals and conditions, underscoring both their promise and their limitations.

Depression

The placebo effect in depression has received considerable attention. Meta-analyses of antidepressant trials show that placebo responses may contribute significantly to symptom improvement, especially in the case of patients with mild to moderate symptoms [21]. Neurobiological research has indicated that placebo interventions can have comparable neural effects to taking antidepressants [22]. However, there are differences among individuals in the degree to which they respond to placebo interventions. A study shows that higher responses were observed in patients with mild or short-duration episodes, and lower responses were observed in those with severe or chronic depression; additionally, patients who previously responded well to antidepressant treatment were more likely to show a robust placebo response [23].

Anxiety Disorders

Placebo effects play a significant role in anxiety disorders, with clinical studies showing meaningful symptom reductions in conditions such as generalized anxiety disorder (GAD) and social anxiety disorder (SAD). A large-scale meta-analysis found that placebo responses were substantial across anxiety and stress-related disorders, although the magnitude of improvement varied between conditions. In particular, GAD demonstrated a strong placebo response, whereas SAD also showed notable improvements, but generally to a lesser extent [24].

Case studies of placebo effects

Successful Clinical Cases

An interesting example of effective placebo treatments comes from the irritable bowel syndrome (IBS). Randomized trials have consistently shown that placebo treatments often improve symptoms as much as active drugs. Since IBS has both physiological and psychological aspects, it is particularly susceptible to placebo effects because of the extent to which both the psychological and the neurobiological mechanisms matter [25]. Another case comes from osteoarthritis of the knee, where a 3-week randomized controlled trial using open-label placebos (OLPs) (in which patients were informed they were taking inert pills) found significantly greater pain reduction compared to a no-treatment control group [26]. The cases described above provide examples of how incorporating placebo principles can improve responses to treatment, particularly when the presenting disorders are those with subjectively defined symptoms.

Failures and Misinterpretations

On the other hand, placebo effects sometimes cause significant confusion. If placebo-induced improvements are mistaken for genuine therapeutic outcomes, patients may stop successful treatments prematurely, causing them more harm and eroding trust in medical treatment [27]. Additionally, high placebo response rates often hide the actual effects of new drugs in clinical trials, resulting in failures of studies and creating considerable barriers to developing drugs and gaining market approval [28].

Limitations of placebo research

Methodological Issues

Designing studies that isolate placebo effects is not straightforward. Ensuring effective blinding is often challenging; participants sometimes figure out if they are receiving the real treatment or a placebo, which can skew results [27]. The diversity of placebo forms complicates the issue further, making standard protocols hard to establish [29]. Choosing the right control groups and outcome measures is especially difficult when dealing with subjective symptoms like pain or mood [30]. 

Variability in Responses

There is wide variability in placebo responses influenced by genetics, psychological factors, environmental influences, and the nature of the disease itself [31]. For example, individuals with certain genetic variants affecting dopamine or opioid receptor activity (e.g., the mu opioid receptor) appear to have their placebo response altered [32]. Similarly, Placebo responses are influenced by individual psychological traits, such as optimism, self-efficacy, and personality characteristics, as well as prior treatment experiences and expectations. Contextual factors, including social environment and cultural background, further modulate these responses. The interplay of these variables results in considerable heterogeneity, making it difficult to predict which individuals will benefit from placebo interventions [33].

Ethical Considerations in Placebo Use

The ethical implications of placebo use are still a significant point of contention in both research and clinical practice. Deception is one of the main issues. With placebo treatment, patients are typically deceived into believing they are receiving an inert treatment, invalidating informed consent, a crucial principle of ethics in medicine [34]. Using deception to elicit healing also raises concerns regarding patient autonomy and trust, even if it is effective [35].

Withholding proven treatment from a control group in a clinical trial is an ethical consideration that could harm participants. This is a bigger issue in research when the study focuses on life-threatening or progressive diseases. Ethical guidelines like the Declaration of Helsinki indicate that placebo use is acceptable only when either there is no other proven intervention to use, or when withholding a treatment wouldn't cause the patient any serious harm [36].

OLPs, in which patients are aware they are receiving a placebo, are an ethical alternative to consider. Evidence from clinical studies, such as the randomized controlled trial in irritable bowel syndrome, shows that participants in the OLP group experienced measurable improvements in symptom severity and global relief compared with no treatment, even though they were fully informed that the pills were inert, with informed consent explicitly obtained before participation. This demonstrates that OLPs can be administered ethically, respecting participants’ right to be informed while still producing beneficial effects [37].

It is essential to balance both the scientific rationale for the clinical trial and the ethical consideration of using a placebo in the trial or treatment. Placebo use must prioritize patient welfare, respect the patient's autonomy, and preserve trust in the healthcare system. Without that trust, even well-intentioned placebo use could compromise both clinical outcomes and public trust [2].

Upcoming trends in placebo studies

Incorporating Placebos into Clinical Practice

Clinicians are beginning to explore ways to leverage placebo effects ethically, including approaches that disclose the use of placebos while still achieving improvements. Incorporating placebos into practice also involves improving care delivery. This may include using placebos alongside standard therapies to reduce medication dosages, implementing them in situations where effective treatments are unavailable, or offering them as supportive interventions for conditions with high subjective symptom burdens, such as chronic pain or irritable bowel syndrome. Importantly, these approaches can be aligned with ethical standards by ensuring that patients are fully informed and by positioning placebo use as part of a broader patient-centered strategy.

As research expands, healthcare systems may increasingly view placebos as legitimate therapeutic tools rather than deceptive practices. Standardized guidelines, clinician training, and further clinical trials will be essential for establishing when and how placebo interventions can be applied most effectively. With these steps, the integration of placebos into medical care has the potential to improve outcomes, reduce reliance on unnecessary medications, and broaden the scope of treatment options available to patients.

Exploring Novel Placebo Mechanisms

In addition to the well-known psychological factors, emerging research highlights biological pathways that may shape placebo effects. One promising direction involves genetics: specific gene variants linked to dopamine and serotonin systems have been shown to influence the degree to which individuals respond to placebo, forming what has been called the “placebome” [31]. Placebos may also engage the immune system. Experimental studies demonstrate that placebo treatments can reduce markers of inflammation, such as pro-inflammatory cytokines, while influencing stress-related physiological pathways [38]. Another exciting area is the microbiome-gut-brain axis. Gut microbial composition has been found to affect mood, pain sensitivity, and stress responses, and it is increasingly suggested that these pathways could intersect with placebo mechanisms [39].

Table 1 shows the summary of the key findings of the narrative review.

**Table 1:** Summary of the key findings of the narrative review

Section	Key points	References
Neurobiological Mechanisms	Placebo activates pain-related brain regions (anterior cingulate cortex, insula, thalamus, periaqueductal gray); triggers endogenous opioid release; dopamine pathways involved in reward/motivation; may influence immune and hormonal function	[5–8]
Psychological Mechanisms	Pavlovian conditioning: learned responses to treatment cues; patient expectations: belief in treatment reduces symptoms; therapeutic engagement: empathy, trust, and communication enhance placebo effects	[8–11]
Types of Placebo Treatments	Placebo pills: inert substances that can reduce pain/mood symptoms; sham procedures: mimic interventions (injections, devices, fake surgery); ritual and context contribute to effect	[12–14]
Clinical Trials and Placebo Controls	Placebo-controlled trials isolate specific drug effects; randomization and blinding reduce bias; allocation concealment and predefined outcomes strengthen validity; adequate sample sizes improve power	[15–17]
Placebo Effects – Pain Management	Chronic pain: analgesia lasts weeks/months; relevant for fibromyalgia, osteoarthritis; effects comparable to healthy individuals. Acute Pain: rapid-onset; measurable pain relief within hours post-procedure; precise duration unclear.	[18–20]
Placebo Effects – Mental Health	Depression: contributes to improvement in mild/moderate cases; neural effects similar to antidepressants; response varies with severity, duration, and prior treatment. Anxiety Disorders: symptom reduction in GAD and SAD; GAD shows a stronger response.	[21–24]
Placebo Case Studies	Successful: IBS: placebo improves symptoms similar to drugs; Knee osteoarthritis: open-label placebos reduce pain more than no-treatment controls. Failures/Misinterpretations: placebo improvements misinterpreted as true treatment, leading patients to stop effective therapies and eroding trust; high placebo responses can also obscure drug effects in trials.	[25–28]
Limitations and Ethical Considerations	Methodological: blinding is challenging; placebo diversity complicates protocols; subjective outcomes are difficult to measure. Variability: genetics, psychology, prior experiences, social/cultural factors; response heterogeneity. Ethics: deception undermines informed consent; withholding proven treatment is harmful; open-label placebos are ethical and effective	[2], [27], [29–37]
Upcoming Trends in Placebo Research	Ethical incorporation into clinical practice: adjunct to standard therapy, disclosure, and patient-centered care. Exploring novel mechanisms: genetics (“placebome”), immune system engagement, microbiome-gut-brain axis	[31], [38–39]

## Conclusions

The placebo effect is far more than a medical curiosity; it demonstrates how the brain, through anticipation, treatment outlook, and surrounding context, can shape physical processes. Once regarded primarily as a methodological nuisance, it is now recognized as a genuine mind-body phenomenon with significant therapeutic relevance. Across domains such as pain and mental health, evidence consistently shows that placebo responses yield real, measurable changes in symptoms. These effects arise through interconnected biological and psychological mechanisms, and expanding research into genetic, immunological, and microbiome-related factors continues to deepen our understanding of the healing process. The central challenge is implementing placebo-informed practices in ways that remain transparent and ethically sound. If reconceived not as deception but as a legitimate element of care rooted in human experience, the placebo effect opens the door to holistic, patient-centered medicine. Such an approach could enhance outcomes, preserve trust, and reduce unnecessary reliance on pharmacological solutions, supporting a broader and more integrative model of healthcare.
